# Validity of a Web-Based 24-Hour Dietary Recall of Energy and Nutrient Intakes in Japanese Adults

**DOI:** 10.3390/nu16234140

**Published:** 2024-11-29

**Authors:** Misako Nakadate, Shunichiro Kobayashi, Junko Ishihara, Ribeka Takachi, Shiori Sugawara, Yuchie Hoshina, Kumiko Kito, Ayaka Kotemori, Sachiko Maruya, Aoi Suzuki, Taku Obara, Mami Ishikuro, Fumihiko Ueno, Aoi Noda, Misato Aizawa, Ippei Takahashi, Yudai Yonezawa, Takahiro Yamashita, Shigenori Suzuki, Keiko Murakami, Shinichi Kuriyama

**Affiliations:** 1 School of Life and Environmental Science, Azabu University, 1-17-71 Fuchinobe, Chuo-ku, Sagamihara-shi 252-5201, Japan; nakadate@azabu-u.ac.jp (M.N.); kotemori@azabu-u.ac.jp (A.K.); 2 Graduate School of Environmental Health, Azabu University, 1-17-71 Fuchinobe, Chuo-ku, Sagamihara-shi 252-5201, Japan; me2309@azabu-u.ac.jp (S.K.); kitou@azabu-u.ac.jp (K.K.); 3 Department of Food Science and Nutrition, Nara Women’s University Graduate School of Humanities and Sciences, Kitauoyahigashimachi, Nara-shi 630-8506, Japan; rtakachi@cc.nara-wu.ac.jp (R.T.); maruya@nagoya-wu.ac.jp (S.M.); waa_suzuki@cc.nara-wu.ac.jp (A.S.); 4 Department of Health and Nutrition, Sendai Shirayuri Women’s College, Honda-Cho, Izumi-ku, Sendai-shi 981-3107, Japan; 5 Department of Nutrition, Sendai Seiyo Gakuin College, 4-5-3, Chuo, Aoba-ku, Sendai-shi 980-0021, Japan; y_hoshina@seiyogakuin.ac.jp; 6 Tohoku Medical Megabank Organization, Tohoku University, 2-1 Seiryo-machi, Aoba-ku, Sendai-shi 980-8573, Japan; obara-t@hosp.tohoku.ac.jp (T.O.); m_ishikuro@med.tohoku.ac.jp (M.I.); uenof@med.tohoku.ac.jp (F.U.); a.noda@megabank.tohoku.ac.jp (A.N.); yudai_yonezawa@kagome.co.jp (Y.Y.); takahiro_yamashita@kagome.co.jp (T.Y.); kuriyama@med.tohoku.ac.jp (S.K.); 7 Graduate School of Medicine, Tohoku University, 2-1 Seiryo-machi, Aoba-ku, Sendai-shi 980-8575, Japan; misato.aizawa.q6@dc.tohoku.ac.jp (M.A.); takahashi.ippei.q1@dc.tohoku.ac.jp (I.T.); 8 Tohoku University Hospital, 1-1 Seiryo-machi, Aoba-ku, Sendai-shi 980-8574, Japan; 9 Diet & Well-Being Research Institute, KAGOME Co., Ltd., 17 Nishitomiyama, Nasushiobara 329-2762, Japan; shigenori_suzuki@kagome.co.jp; 10 International Research Institute of Disaster Science, Tohoku University, 2-1 Seiryo-machi, Aoba-ku, Sendai-shi 980-8573, Japan

**Keywords:** dietary assessment tool, validity, 24 h dietary recall, web-based dietary assessment, online dietary assessment

## Abstract

Recently, web-based dietary assessment tools for the targeted population have been developed and used to estimate the dietary intake level in several epidemiological studies. This study aimed to examine the validity of estimating energy and nutrient intake by the web-based 24 h dietary recall (Web24HR), which we developed for the Japanese population. Overall, 228 adults aged ≥20 years who agreed to participate were included. Web24HR was administered three times per person: twice within 3 weeks and once 3 months later. The data on 3-day weighed food records (WFR) at 3-month intervals in the four seasons were collected using the reference method. The intake of energy and nutrients between Web24HR and WFR were compared using Pearson’s correlation coefficients and the Bland–Altman analysis. As results, the correlations were moderate for both men (median r = 0.51) and women (median r = 0.38) except for iodine, retinol, retinol equivalents, and β-tocopherol. The Bland–Altman method revealed that the bias in intake was within ±10% for most nutrients, except for cholesterol, iodine, vitamin C, and the water content, in both sexes. Additionally, monounsaturated fatty acids in men and β-cryptoxanthin in women exhibited an underestimation of more than 10%. In conclusion, the Web24HR intake assessment showed moderate correlations for most nutrients in both sexes. The bias in intake was within ±10% for most nutrients, but there were discrepancies for some nutrients. This tool’s performance is comparable to Japan’s standard dietary exposure assessment methods and will be helpful for future applications in epidemiological studies, though caution is needed for certain nutrient assessments.

## 1. Introduction

Accurate and often repeated measures of diet are needed for a robust assessment of the relationship between diet and health in population-based studies [1]. Several dietary assessment methods, each with advantages and limitations, are selected based on the research objectives and target populations. One method is the 24 h dietary recall (24HR), in which an interviewer questions the participant on their food intake over the past 24 h, starting from the previous day or at the time [2]. This method is relatively easy to administer; however, assessing habitual dietary intake with a single data collection is challenging, and multiple data collections may be required. Therefore, its application in large-scale epidemiological studies may be difficult. Information and communication technology advancements have recently led to the development of web-based 24HR (Web24HR) [3], primarily in Western countries (Automated Self-Administered 24 h recall [ASA24], developed by the National Cancer Institute in the United States [4]; INTAKE24 [5]; and Compl-EAT [6]). These have been adapted in various countries and life stages and used in large-scale epidemiological studies. Systematic reviews have reported that the usefulness of these tools is comparable to existing methods, and it is expected that tools utilizing these technologies will become mainstream [7,8,9].

In Japan, the weighed food record (WFR) used in the National Health and Nutrition Survey (NHNS), has been used as a standard method for dietary assessments. Unlike the 24HR commonly used in other countries, the WFR is preferred for its detailed accuracy; however, this method is labor-intensive, particularly for responders in multi-member households or working ages, as they must accurately report the dietary intake of each household member. This is also a time-consuming process for investigators who need to verify the accuracy of the recorded data [10]. In Japan and some Asian countries, mixed dishes are commonly consumed as part of the traditional diet [11,12]. Additionally, a growing trend toward the consumption of ready-made foods has been reported in many countries [13,14,15], including Japan, where this trend has been steadily increasing [15]. Considering the diversity of eating habits across the region, particularly in Asia, developing dietary assessment tools that are based on dishes rather than individual ingredients is crucial [16]. A recipe database of mixed dishes that are consumed typically in the Japanese diet were necessary for the adaptation of the Web24HR for the Japanese population. However, to our knowledge, a comprehensive database of recipes for mixed dishes has not yet been developed in Japan. Therefore, a standardized recipe database for mixed dishes based on Japanese observational study data was developed and integrated into the Web24HR system [17]. The system, called “Automated Web-based Assessment System using Recipe Data for Japanese (AWARDJP)”, was designed as a dietary assessment tool for the Japanese population in epidemiological studies. Although its applicability and feasibility for large-scale epidemiological studies have been examined [18], its validity for estimating dietary intake has not yet been evaluated. We aimed to evaluate the validity of the measurement of habitual energy and nutrient intake using the AWARDJP with the WFR as the reference method for comparison.

## 2. Materials and Methods

### 2.1. Study Setting and Participants

This study was part of the Tohoku Medical Megabank Project (TMM) conducted by Tohoku University, Tohoku Medical Megabank Organization (ToMMo), and Iwate Medical University, Iwate Tohoku Medical Megabank Organization [19]. This project was established to promote the reconstruction of the Tohoku region [20] and address medical problems in the aftermath of the Great East Japan Earthquake and resulting tsunami that occurred on 11 March 2011, which included the TMM Community-Based Cohort Study and the TMM Birth and Three-Generation Cohort Study. These cohort studies included a food frequency questionnaire (FFQ) for adults, and this study was conducted as a sub-study of the FFQ validity study [20]. The eligibility criteria of the study were residents aged ≥ 20 years residing in Miyagi Prefecture who were able to visit either Sendai, Iwanuma, or Ishinomaki community support centers, which the ToMMo established as local facilities for voluntary admission-type recruitment and health assessments of participants [20]. Pregnant individuals were excluded. Those wishing to participate were fully informed, in writing and orally, of the study purpose and content, emphasizing the voluntary nature of their participation. Consent was obtained from 228 participants in the study. As an incentive, participants received rewards and were provided with a report of their energy and nutrition consumption based on the 12-day WFR. This research underwent a comprehensive scientific and ethical review by the Ethics Committee of ToMMo (2019-4-027) and all other collaborating research institutions.

### 2.2. Study Design and Data Collection

**Figure 1:**
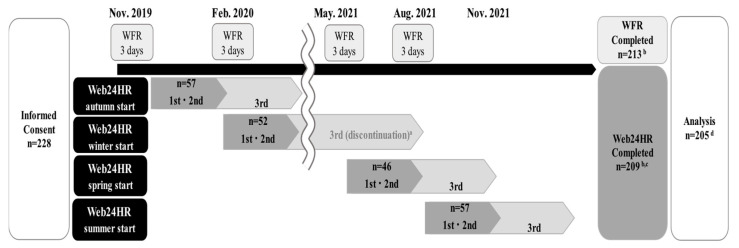
Study scheme. ^a^ Owing to the impact of the epidemic of COVID-19 infection in 2020, the study scheduled for May 2020 or later was postponed for one year, and the third for the winter-start group of Web24HR was cancelled. ^b^ Three individuals declined to participate in the study prior to its implementation and 12 declined to participate during the study period. ^c^ One individual declined to participate in the Web24HR only, and 3 did not respond to Web24HR. ^d^ Two participants who were unable to complete the Web24HR twice within 3 weeks of WFR implementation were excluded. WFR, 12-day weighed food record; Web24HR, the AWARDJP by web-based 24 h recall dietary assessment method.

The study scheme is shown in Figure 1. Upon obtaining consent, participants received lifestyle questionnaires and dietary assessment items. The study was conducted between November 2019 and November 2021, with assessments performed every 3 months in each season at each community support center (Sendai, Iwanuma, and Ishinomaki in Miyagi Prefecture) or online. Lifestyle questionnaires were administered annually, and the collection of WFR occurred over three consecutive days (two weekdays and one holiday) during four seasons (12 days, 1 year). The collection of Web24HR was randomly categorized into four household groups for each season and conducted three times for each group: non-consecutive on two days within 3 weeks after the WFR, and 1 day after 3 months. Owing to the impact of the coronavirus disease (COVID-19) pandemic in 2020, the study scheduled for May 2020 or later was postponed for 1 year, and the third for the winter-start group of Web24HR was cancelled. Among the 228 participants from whom consent was obtained, three declined to participate before the study, and 12 declined during the study period. One declined only for Web24HR, three did not respond to Web24HR, and 209 responded, including those who had forgotten more than once to complete Web24HR. Among those who completed the study, 205 were included in the analysis, excluding four who could not complete the Web24HR twice. A total of 10.1% of participants had their data excluded.

### 2.3. Web-Based 24 H Recall by AWARDJP

AWARDJP was developed to follow the Automated Multiple-Pass Method (AMPM), a standard procedure for the 24HR developed by the United States Department of Agriculture [21,22]. The procedure was slightly modified for the Japanese population, and a user interface was developed to ensure that the responders can self-administer it by simply following instructions on the screen for the selection of the name of the dishes and the cooking details [18]. Participants were notified in a specific period (within 3 weeks after the interview of WFR), during which they would receive a request via e-mail to enter the dietary intake of previous 24 h in the AWARDJP. Upon receiving the email, the participants independently accessed the AWARDJP using the specified URL and login information. If the entry was not confirmed on the day, a reminder email was sent the following day. If there was no response, another reminder was sent by email 1 week before the period expired.

The participants could also choose the interviewer-administered method over the telephone, depending on their ability and availability to use a personal computer (PC) at home. A full-size dish scale was distributed to all participants in advance. The interviewers were trained to use them to confirm portion sizes [18].

### 2.4. Weighed Food Records

The study was conducted in accordance with the same procedures as the semi-weighted household dietary record used in the NHNS [23]. The study details of WFR are described elsewhere [20]. In brief, 12-day WFR data were collected for 2 weekdays and a weekend day every 3 months to cover all seasons. The participants were instructed on how to weigh and record their diet before they started the WFR. They were asked to measure the food items before cooking using the provided cups, spoons, and digital scales (Tanita Co. Ltd., Tokyo, Japan) when cooking at home. For processed or cooked food, such as meals from restaurants, participants were requested to record product information and the approximate amount consumed. The recorded details were later verified by dietitians, who were trained based on a standardized manual, through face-to-face or online interviews.

### 2.5. Estimation of Energy and Nutrient Intake

Energy and nutrient intake from Web24HR and WFR were calculated by assigning food codes according to the Standard Tables of Food Composition in Japan 2010. The intake of energy and 53 nutrients includes the nutrients listed in Japan’s Standard Tables of Food Composition in 2010 [24], n-3/n-6 polyunsaturated fatty acids [25], and ethanol.

### 2.6. Statistical Analysis

To determine the validity of intake by Web24HR, Pearson’s correlation coefficients (CCs) between intakes based on the 12 d WFR and Web24HR were calculated using energy-adjusted intake by the residual method. The mean difference between the methods (bias: Web24HR− WFR) and the 95% confidence interval (95% CI) for bias were calculated using the Bland–Altman method. Additionally, the 95% limits of agreement (LOAs) were calculated the range of differences between these methods to assess agreement [26,27]. The agreement of log-transformed intake data was interpreted by taking their antilogs. The antilog interpretation leads to a dimensionless ratio, which allows us to express agreement as an intuitively understandable value [26]. That is, we have used the logarithmic property to replace the difference in logarithms with a ratio: logA − logB = log(A/B). Therefore, to align the interpretation with previous studies, the mean difference can be interpreted as a ratio when antilogs are obtained. The bias of the log-transformed values was assessed by taking the antilog and multiplying by 100, yielding a ratio for evaluating over- or underestimation. A result of 100% indicates no difference, while values above or below 100% suggest over- or underestimation, respectively (e.g., 110% indicates overestimation by 10% and 90% indicates underestimation by 10%). If the bias from the Bland–Altman method is ±10% or more and the interval estimate is significant, it is a fixed error. If the regression slope in the regression line of differences is significantly different, it is a proportional error [28,29]. The regression slope (β) was calculated using the mean of both methods as independent variable and the difference between the methods as dependent variable. When both the dependent and independent variables were expressed in natural logarithms, the slope is expressed as a percentage change (β%), indicating that a 1% increase in the independent variable corresponds to an increase in the dependent variable by the percentage value of the slope. The LOA range is one-half to twice, which is within 50–200%, indicating an acceptable error [29]. The normality of all intake distributions was assessed using the Shapiro–Wilk test. For example, 3 d Web24HR intakes were found to be non-normally distributed, except for the ash, copper, molybdenum and β-tocopherol contents in women. All analyses used log-transformed intakes.

The data used in the analysis were 2 or 3 days of Web24HR (3-d Web24HR) and 12 days of WFR (12-d WFR) data collected during the entire study period without violating the exclusion criteria for analysis. Some Web24HR data included only 2 days of data (n = 50 and 24% of all analyzed participants). All analyses are presented separately for men and women, and the results for all participants are presented in the Supplementary Materials for comparison with other studies (Tables S3–S7). Statistical analyses were performed using SAS Ver. 9.4 (SAS Institute Inc., Cary, NC, USA).

## 3. Results

Participants’ characteristics at baseline are presented in Table 1. More than half of the participants were women (n = 122, 59.5%). The mean (standard deviation [SD]) age was 53.6 (15.8) years, with men being slightly older (55.7 (16.2) years) than women (52.1 (15.4) years). A higher proportion of men chose to complete the self-administered method (self-administered, 58.8%; interviewer-administered, 38.8%), while the proportion of women was equal for both methods. The mean body mass index (BMI) was 23.2 (3.6) kg/m^2^, with women having a slightly lower BMI than men (men: 23.9 (3.0) kg/m^2^; women: 22.6 (3.9) kg/m^2^). The proportion of smokers was low (men: 22.4%, women: 1.6%).

The Pearson’s CCs between the intakes in the 3 d Web24HR and the 12 d WFR, along with the Bland–Altman method, are presented in Table 2 and Table 3. The median CCs by sex showed higher values for men than women, but both were above moderate. The median CCs (minimum to maximum) were r = 0.51 (0.06–0.87) for men and r = 0.38 (0.09–0.72) for women. Nutrients that showed CCs < 0.2 in both men and women were iodine, retinol, retinol equivalents, and β-tocopherol. The results of the Bland–Altman method showed that few nutrients had a bias of within ±10% (46/53 nutrients within a ±10% bias for men and 47/53 nutrients for women), while over half of the nutrients had proportional errors. As a sensitivity analysis, the energy and nutrient intakes from the 2 d Web24HR and 3 d WFR were assessed in the same season. Although a few nutrients showed proportional errors, the CCs and fixed errors were similar and comparable (Tables S1–S4).

Among the nutrients contributing to energy production, for women, protein (with a bias of 108.1 [103.1–113.3]) and carbohydrates (106.8 [101.3–112.6]) were overestimated. Cholesterol showed a CC of more than moderate and was statistically overestimated by more than 10% in the estimated intake for both sexes. For men, the CC was r = 0.52 with a bias [95% CI] of 114.6 [101.8–129.1]. For women, the CC was r = 0.38 with a bias [95% CI] of 115.2 [105.7–125.5]. Alternatively, the nutrients that were underestimated by both sexes were water (men: r = 0.21, 72.8 [67.1–78.8]; women: r = 0.44, 71.9 [68.3–75.8]) and vitamin C (men: r = 0.66, 85.7 [76.7–95.6]; women: r = 0.67, 88.2 [81.9–94.9]). Proportional errors were found for cholesterol and water in both men and women, and for vitamin C only in women. The LOAs for these nutrients were wide. In men, monounsaturated fatty acids (r = 0.48, 89.5 [81.5–98.3]) and, in women, β-cryptoxanthin (r = 0.34, 76.6 [59.7–98.3]) exhibited underestimation, and the degree of bias was substantial. Proportional errors were observed for both nutrients. The CCs for iodine were small, and the bias was significant (men: r = 0.18, 53.4 [34.0–83.7], women: r = 0.15, 42.0 [29.5–59.8]).

## 4. Discussion

The validity of energy and nutrient intake estimations by Web24HR using AWARDJP was moderately correlated for both sexes, except for iodine, retinol, retinol equivalents, and β-tocopherol. The bias in intake was within ±10% for most nutrients, except for cholesterol, iodine, vitamin C, and water in both men and women. Systematic errors were observed for protein and carbohydrates in women, but the magnitude was small (Table S8). The results were similar to those reported in previous studies in which the validity of Web24HR was evaluated [30,31,32,33,34,35]. Web24HR using AWARDJP is considered a valid method for estimating energy and nutrient intakes. Several studies have reported improved CCs with an increase in the number of days [36,37], which is consistent with the finding of this study. This may be because, as with the existing face-to-face-based 24HR, multiple days are preferred. However, some nutrients exceed the proportional errors and the range of LOAs, suggesting that the influence of within-person or between-person variability cannot be excluded.

Cholesterol intake in Web24HR correlated with reference method moderately for both sexes but was overestimated by approximately 10%. The validity studies of the tool myfood24 tended to underestimate cholesterol intake compared to the face-to-face-based 24HR [33], and a Japanese study using a web-based FFQ also reported similar results [38]. Additionally, more days are needed for Japanese adults to determine their habitual cholesterol intake compared to other nutrients [39]. The WFR data from this population showed that within-individual variation contributed significantly to the observed dietary variability (73.8% for men and 80.3% for women). Additionally, the results of the deattenuated analysis indicated an increase in CCs (energy adjustment (Table 2 and Table 3): r = 0.52, deattenuated (Table S9): r = 0.58 for men and r = 0.38, r = 0.45 for women), suggesting improved alignment between the Web24HR and reference methods [39]. The number of days required to capture habitual intake, based on the obtained intra-individual variation, was approximately 30 days, which is consistent with a previous study.

Conversely, vitamin C and water contents were underestimated by more than 10%, despite the moderate correlation. In similar studies assessing errors in vitamin C, the absolute error was small (AWARDJP (Table S3): −10.1 mg, R24W [31]: −43.1 mg, Foodbook24 [35]: −14.4 mg). The result for the water content showed the opposite trend to that of previous studies, which reported an overestimation [31]. The AMPM method, which was the basis of AWARDJP, has a procedure to confirm any omissions [40]. However, this may be under-reporting, especially if a participant consumed one bottled beverage several times per day. Considering a slightly lower CC for men, the possibility cannot be denied that they forgot to declare beverages. Recently, a reduction in total water content and mortality risk has been reported [41,42]. These intakes need to be evaluated in the future.

Additionally, iodine intake was considered particularly difficult to measure in the present results. In Japan, the primary source of iodine is algae (60%), followed by soup stock (30%) [43], and the food group intake of algae in this study showed the same underestimation as that for iodine (r = 0.41, bias [95% CI]; 63.9 [51.8–78.8] for men and r = 0.33, 63.3 [51.8–77.4] for women, Table S5). In a large epidemiological study in Japan, the assessment estimation derived from the web-based FFQ showed comparable results for assessing iodine intake [38], although algae was consumed less frequently than other food groups, and the actual amount consumed was also small. Iodine has a wide range of 95% LOAs, and each dietary evaluation method may not be used on the same day, or the frequency of intake may affect measurement errors. The analysis of the percentage contributions of variance components within individuals revealed that iodine was largely influenced by within-person variation (86.1% in men and 96.4% in women). Notably, the contribution rate of algae intake was consistent with previous research, suggesting that the daily intake frequency of algae, a significant iodine contributor, may affect the results (this study: 90.8% for men and 86.6% for women; previous study [44]: 94.1% for men and 93.8% for women). Additionally, the results of the deattenuated analysis indicated an increase in CCs: iodine (Table 2 and Table S9) improved from r = 0.18 to r = 0.23 in men and from r = 0.15 to r = 0.24 in women; algae (Tables S5 and S10) improved from r = 0.41 to r = 0.56 in men and from r = 0.33 to r = 0.41 in women.

The strength of this study is that it comprehensively examined many nutrients by conducting Web24HR using AWARDJP and comparing the validation of intakes with WFR. While certain nutrients may not have been quantitatively evaluated in this study, the data gathered here can serve as foundational material for future research into individual nutrients. In the future, it may be necessary to examine the validity using objective indicators, such as biomarkers. This study has certain limitations. First, the intake obtained by Web24HR may not reflect long-term intake of some of the nutrients and foods through the 3-day administration. Additionally, reproducibility of the Web24HR could not be assessed due to the study design. The number of days required to assess long-term intake depends on the type of nutrient [39,44,45], and we based the days on prior studies that considered the number of days required to assess habitual energy intake in Japanese populations. In future studies, longer and repeated administration of Web24HR should be included. Second, the target population was relatively health-conscious, and the area was limited; it may not represent the general adult population. Considering the effects of regional differences, this study recruited residents from urban and rural areas within the same region; however, many validation studies of dietary assessment methods adopt similar schemes or study settings, and biases affecting validity are not different when comparing the diets of the same participants. Third, changes in eating habits caused by cancellations or refraining from participating in research due to the COVID-19 pandemic may have impacted the population. However, even in cases where the study was postponed, the results indicated that numerous nutrients had a low bias and moderate or higher CCs. Finally, there was potential bias introduced by allowing participants to choose between self-administered and interviewer-administered Web24HR methods (Tables S11–S18). Older participants were more likely to choose the interviewer-administered method (Percentage of people aged 60 and over by method; self-administered 24.3%, interviewer-administered 57.4%), suggesting possible selection bias. In the self-administered group, especially among women, several micronutrients, such as iodine and sodium showed low CCs (r < 0.2). While other nutrients did not show significant differences between methods, this inconsistency may have affected results, particularly for certain micronutrients. This should be considered when interpreting the findings.

## 5. Conclusions

The Web24HR assessment of energy and nutrient intake showed moderate correlations for both sexes, except for iodine, retinol, retinol equivalents, and β-tocopherol. The bias in intake was generally within ±10% for most nutrients, with exceptions, such as cholesterol, iodine, vitamin C, and water. Although these nutrients showed bias in intake, a consideration of within-person variation may help address these issues. Further quantitative validation using biomarkers may also be necessary. However, since the tool demonstrated performance comparable to Japan’s standard dietary exposure assessment method, it contributes to the development of epidemiological studies, helping to explore the link between a broader range of dietary intakes and health outcomes. Care should still be taken when interpreting the intake assessments for some nutrients.

## Figures and Tables

**Table 1:** Characteristics of the participants.

		Overall (n = 205)		Men (n = 83)		Women (n = 122)		*p*-Value ^c^
Age, years, mean (SD)		53.6	(15.8)	55.7	(16.2)	52.1	(15.4)	0.1070
Body mass index, kg/m^2^, mean (SD) ^a^		23.2	(3.6)	23.9	(3.0)	22.6	(3.9)	**0.0121**
Current smoker, number (%)		21	(10.2%)	19	(22.4%)	2	(1.6%)	**0.0100**
Eating out at least once a week, number (%)		86	(42.0%)	36	(42.4%)	50	(41.0%)	0.1206
Prepared foods consumed at least once a week, number (%)		64	(31.2%)	27	(31.8%)	37	(30.3%)	0.5800
Collection method by Web24HR, number (%)	self-administered	111	(54.1%)	50	(58.8%)	61	(50.0%)	0.1486
	interviewer-administered	94	(45.9%)	33	(38.8%)	61	(50.0%)	
Web24HR’s season of	spring	45	(22.0%)	21	(25.3%)	24	(19.7%)	0.6750
implementation, number, (%) ^b^	summer	57	(27.8%)	23	(27.1%)	34	(27.9%)	
	autumn	56	(27.3%)	23	(27.7%)	33	(27.0%)	
	winter	47	(22.9%)	16	(18.8%)	31	(25.4%)	

SD, standard deviation; Web24HR, uses the AWARDJP by web-based 24-h dietary recall. ^a^ Missing data on weight (n = 2). ^b^ The Web24HR was randomly divided into four groups of households for each season of implementation. ^c^ Age and body mass index were analyzed using an independent *t*-test, while other variables were analyzed using the chi-squared test, and both analyses were based on sex (bold is *p* < 0.05).

**Table 2:** Pearson’s correlation coefficients and comparisons using the Bland–Altman method between the energy and nutrient intakes of each dietary exposure assessment method and the 3 d Web24HR using the 12 d WFR as a standard (Men).

		3d Web24HR Intake ^a^						12d WFR Intake ^b^						Pearson’s CC Energy Adjustment		Bland Altman Method ^c, d^				
		Crude			Energy Adjustment			Crude			Energy Adjustment									
		Mean	(SD)	Median	Mean	(SD)	Median	Mean	(SD)	Median	Mean	(SD)	Median			Bias, %(95%CI)		95% LOA, %	β	
***Men* (*n* = *83*)**																				
Energy	(kcal)	2330	(838)	2196	2330	(838)	2196	2247	(461)	2171	2247	(461)	2171	0.40	**	100.4	(93.9, 107.4)	54.9, 183.5	0.57	**
Water content	(g)	1788.3	(702.5)	1680.1	1759.4	(741.1)	1680.2	2396.2	(665.0)	2339.5	2351.9	(450.8)	2319.4	0.21		72.8	(67.1, 78.8)	35.4, 149.5	0.34	*
Protein	(g)	86.2	(31.0)	80.6	82.6	(14.7)	81.4	80.5	(19.9)	77.0	78.9	(11.6)	77.9	0.50	**	104.1	(96.9, 111.9)	54.6, 198.7	0.43	**
The sum of amino acid residues	(g)	25.7	(9.2)	23.9	25.2	(7.1)	24.4	28.3	(9.9)	27.5	27.7	(7.5)	26.6	0.36	**	91.0	(84.5, 97.9)	46.9, 176.4	−0.03	
Total fat	(g)	69.3	(32.1)	62.0	64.5	(12.6)	65.7	71.3	(18.7)	68.1	69.7	(11.6)	70.3	0.40	**	92.1	(84.4, 100.7)	41.7, 203.6	0.57	**
Saturated fatty acids	(g)	20.43	(11.43)	17.47	18.69	(4.60)	18.25	20.28	(5.66)	20.13	19.88	(3.92)	20.26	0.33	**	93.5	(85.0, 102.9)	39.6, 221.1	0.67	**
Monounsaturated fatty acids	(g)	24.87	(12.43)	22.83	23.12	(5.77)	23.36	26.31	(7.63)	25.93	25.70	(5.23)	25.85	0.48	**	89.5	(81.5, 98.3)	38.7, 207.2	0.53	**
Polyunsaturated fatty acids	(g)	14.27	(5.98)	12.90	13.65	(3.66)	13.21	14.82	(4.42)	13.90	14.48	(2.83)	14.46	0.37	**	93.3	(86.0, 101.1)	45.0, 193.3	0.44	**
n-3 polyunsaturated fatty acids	(g)	2.6	(1.5)	2.2	2.5	(1.3)	2.1	2.5	(1.1)	2.4	2.5	(0.8)	2.4	0.56	**	96.8	(89.7, 104.3)	49.2, 190.1	0.40	**
n-6 polyunsaturated fatty acids	(g)	11.6	(5.1)	10.1	11.1	(3.1)	10.8	12.1	(3.5)	11.5	11.8	(2.4)	11.7	0.33	**	92.7	(85.4, 100.7)	44.2, 194.5	0.48	**
Triacylglycerol equivalents	(g)	61.7	(29.1)	55.8	57.4	(11.9)	57.6	63.0	(16.9)	61.2	61.5	(10.3)	62.4	0.43	**	92.7	(84.7, 101.4)	41.4, 207.3	0.57	**
Cholesterol	(mg)	453	(261)	402	421	(167)	390	362	(126)	351	355	(102)	342	0.52	**	114.6	(101.8, 129.1)	39.4, 333.2	0.56	**
Carbohydrate	(g)	305.3	(128.3)	279.3	289.1	(51.4)	299.2	290.2	(71.8)	276.1	284.1	(36.7)	287.9	0.75	**	100.7	(93.9, 108.1)	53.5, 189.8	0.53	**
Total dietary fiber	(g)	17.2	(7.9)	16.3	16.6	(5.7)	15.7	15.9	(5.8)	15.3	15.7	(5.0)	14.7	0.63	**	104.6	(96.1, 113.7)	49.2, 222.4	0.27	*
Soluble dietary fiber	(g)	4.2	(1.9)	3.9	4.0	(1.3)	3.8	3.8	(1.4)	3.6	3.8	(1.3)	3.4	0.50	**	104.9	(97.0, 113.5)	51.8, 212.4	0.34	*
Insoluble dietary fiber	(g)	12.4	(5.9)	11.5	12.0	(4.4)	11.3	11.5	(4.4)	10.8	11.3	(3.7)	10.7	0.66	**	105.1	(97.2, 113.6)	52.1, 211.8	0.21	
Ash content	(g)	19.6	(6.0)	18.9	19.1	(3.9)	18.9	19.5	(4.7)	19.6	19.2	(3.2)	19.0	0.57	**	98.8	(92.9, 105.1)	56.8, 171.9	0.27	
Salt equivalent	(g)	10.8	(3.4)	10.2	10.6	(2.7)	10.2	10.9	(2.8)	10.5	10.8	(2.2)	10.6	0.41	**	97.7	(91.6, 104.2)	54.9, 173.9	0.23	
Sodium	(mg)	4268	(1327)	4060	4197	(1056)	4023	4315	(1113)	4135	4255	(865)	4157	0.42	**	97.6	(91.0, 104.8)	51.9, 183.7	0.22	
Potassium	(mg)	2906	(1074)	2590	2807	(700)	2749	2875	(968)	2716	2817	(751)	2678	0.72	**	99.9	(93.0, 107.4)	52.5, 190.3	0.12	
Calcium	(mg)	623	(316)	540	593	(226)	558	580	(198)	567	569	(163)	541	0.62	**	100.7	(91.9, 110.2)	44.5, 227.5	0.44	**
Magnesium	(mg)	312	(107)	282	303	(72)	298	301	(89)	289	295	(66)	285	0.66	**	102.4	(95.9, 109.3)	57.1, 183.6	0.13	
Phosphorus	(mg)	1253	(442)	1118	1205	(231)	1201	1189	(298)	1156	1166	(188)	1149	0.55	**	102.8	(95.9, 110.1)	55.3, 190.9	0.34	*
Iron	(mg)	9.7	(4.1)	8.9	9.3	(2.8)	8.9	8.9	(2.7)	8.5	8.7	(2.0)	8.5	0.56	**	105.2	(97.8, 113.3)	54.3, 203.8	0.36	**
Zinc	(mg)	10.1	(4.0)	9.1	9.6	(1.9)	9.3	9.3	(2.3)	9.2	9.1	(1.2)	9.0	0.41	**	104.8	(98.3, 111.9)	58.6, 187.6	0.49	**
Copper	(mg)	1.41	(0.53)	1.28	1.37	(0.30)	1.36	1.31	(0.36)	1.30	1.30	(0.24)	1.28	0.54	**	103.1	(99.0, 107.3)	71.8, 147.8	0.38	**
Manganese	(mg)	3.70	(2.50)	3.13	3.59	(2.02)	3.25	3.50	(1.29)	3.23	3.44	(0.98)	3.29	0.56	**	100.6	(94.4, 107.3)	56.8, 178.3	0.35	**
Iodine	(μg)	731	(1636)	155	650	(1381)	148	1108	(2293)	245	1063	(2043)	224	0.18		53.4	(34.0, 83.7)	0.9, 3028.5	0.07	
Selenium	(μg)	56	(30)	51	54	(25)	49	59	(23)	57	58	(21)	54	0.22	*	89.3	(78.7, 101.4)	28.6, 279.3	0.50	**
Chromium	(μg)	8	(6)	7	7	(3)	6	7	(2)	7	7	(2)	7	0.33	**	102.0	(91.2, 114.1)	37.4, 278.2	0.81	**
Molybdenum	(μg)	201	(81)	190	196	(65)	183	194	(72)	184	189	(53)	187	0.55	**	102.2	(95.3, 109.7)	54.3, 192.7	0.11	
Retinol	(μg)	307	(509)	200	293	(497)	182	237	(203)	199	235	(214)	183	0.08		105.0	(84.7, 130.3)	15.2, 726.5	0.51	*
Retinol equivalents	(μg)	661	(577)	527	641	(538)	542	560	(287)	482	551	(271)	474	0.20		106.9	(91.5, 124.8)	26.6, 429.6	0.48	**
α-Carotene	(μg)	493	(368)	382	486	(338)	409	406	(213)	379	404	(204)	384	0.21		103.9	(84.6, 127.5)	16.5, 653.8	0.76	**
β-Carotene	(μg)	3765	(2522)	3585	3696	(2376)	3289	3029	(1807)	2598	2991	(1654)	2594	0.50	**	109.8	(93.8, 128.6)	26.6, 453.3	0.52	**
β-Cryptoxanthin	(μg)	226	(465)	76	227	(493)	68	194	(250)	84	191	(243)	91	0.44	**	79.4	(59.5, 105.9)	6.0, 1057.1	0.25	
β-Carotene equivalents	(μg)	4170	(2692)	3939	4091	(2509)	3696	3608	(2164)	3019	3551	(1960)	2990	0.53	**	105.2	(90.6, 122.1)	27.7, 399.8	0.43	**
Vitamin D	(μg)	9.2	(8.8)	6.2	8.9	(7.8)	6.1	8.2	(5.5)	6.9	8.1	(5.6)	6.9	0.54	**	94.2	(80.7, 109.8)	23.7, 374.6	0.41	**
α-Tocopherol	(mg)	8.7	(4.2)	7.4	8.3	(2.9)	7.7	8.2	(2.8)	7.8	8.1	(2.2)	7.4	0.58	**	99.7	(91.4, 108.6)	46.0, 215.9	0.53	**
β-Tocopherol	(mg)	0.5	(0.3)	0.4	0.5	(0.2)	0.5	0.4	(0.1)	0.4	0.4	(0.1)	0.4	0.06		101.5	(98.0, 105.2)	73.7, 139.9	1.06	**
γ-Tocopherol	(mg)	12.3	(5.9)	10.5	11.8	(4.3)	10.8	12.2	(3.9)	11.8	11.9	(2.7)	12.0	0.35	**	96.2	(87.6, 105.5)	41.8, 221.4	0.48	**
δ-Tocopherol	(mg)	3.1	(1.5)	2.8	3.0	(1.2)	2.8	3.1	(1.1)	3.1	3.1	(0.8)	2.9	0.26	*	96.2	(88.8, 104.3)	46.6, 198.6	0.44	**
Vitamin K	(μg)	328	(227)	263	321	(203)	283	283	(148)	254	277	(130)	238	0.59	**	106.7	(94.5, 120.5)	35.8, 317.9	0.35	**
Vitamin B1	(mg)	1.10	(0.46)	1.03	1.08	(0.29)	1.07	1.14	(0.31)	1.11	1.13	(0.21)	1.10	0.31	**	97.3	(93.0, 101.8)	64.9, 145.9	0.52	**
Vitamin B2	(mg)	1.51	(0.64)	1.29	1.47	(0.39)	1.44	1.46	(0.43)	1.44	1.45	(0.32)	1.43	0.64	**	100.5	(95.9, 105.3)	65.8, 153.5	0.42	**
Niacin	(mg)	20.4	(8.0)	19.6	19.8	(5.9)	20.1	20.6	(6.4)	19.8	20.3	(5.0)	19.9	0.59	**	96.3	(89.5, 103.7)	49.6, 187.2	0.30	*
Vitamin B6	(mg)	1.47	(0.56)	1.40	1.44	(0.42)	1.44	1.43	(0.51)	1.30	1.41	(0.39)	1.35	0.64	**	101.0	(96.8, 105.3)	69.3, 147.2	0.10	
Vitamin B12	(μg)	8.2	(7.4)	6.0	7.8	(6.2)	6.2	7.5	(4.4)	6.3	7.3	(4.2)	6.5	0.51	**	95.1	(82.9, 109.0)	27.9, 324.4	0.43	**
Folate	(μg)	352	(144)	324	344	(116)	334	370	(156)	339	362	(131)	340	0.61	**	95.5	(88.0, 103.8)	45.5, 200.7	−0.03	
Pantothenic acid	(mg)	7.22	(2.32)	7.02	7.01	(1.24)	6.98	6.89	(1.81)	6.72	6.77	(1.14)	6.70	0.51	**	103.0	(97.0, 109.3)	60.2, 176.2	0.22	
Biotin	(μg)	30.5	(13.2)	27.1	29.7	(10.6)	29.0	31.5	(11.1)	29.1	31.0	(9.8)	29.5	0.58	**	94.5	(87.1, 102.5)	45.4, 196.4	0.26	*
Vitamin C	(mg)	95	(46)	89	93	(43)	89	110	(59)	99	108	(51)	99	0.66	**	85.7	(76.7, 95.6)	31.9, 230.2	0.08	
Ethanol	(g)	16.3	(25.1)	4.6	16.3	(25.0)	4.7	14.2	(18.5)	4.9	13.9	(18.0)	4.1	0.87	**	98.1	(82.5, 116.7)	20.7, 465.8	0.06	
**Median (minimum to maximum)**														**0.51**	**(0.06~0.87)**					

SD, standard deviation; Pearson’s CC, Pearson’s correlation coefficient; bias, the mean difference between methods; 95% CI, 95% confidence interval; LOA, 95% limit of agreement [mean difference ± 1.96 *(standard deviation of difference)], expressed as *p* values *: *p* < 0.05 and **: *p* < 0.01. ^a^ 3 d Web24HR: uses the AWARDJP with the web-based 24-h dietary recall survey method. In the entire study period the Web24HR data were collected two or three times. ^b^ 12 d WFR: 12 days of weighted dietary records collected during the entire study period of a year. ^c^ Exponential transform [mean(Web24HR − WFR)] as a ratio of the WFR (all dietary intake data were log-transformed). e.g., 110% indicates overestimation by 10%, and 90% indicates underestimation by 10%. ^d^ The regression slopes of the means of both methods show the differences between the two methods. *p*-values for the the regression slope are shown. When the response and explanatory variables are natural logarithms, a 1% increase in the explanatory variable increases the response variable by β%.

**Table 3:** Pearson’s correlation coefficients and comparisons using the Bland–Altman method between the energy and nutrient intakes of each dietary exposure assessment method and the 3 d Web24HR using the 12 d WFR as a standard (Women).

		3d Web24HR Intake ^a^						12d WFR Intake ^b^						Pearson’s CC Energy Adjustment		Bland Altman Method ^c,d^				
		Crude			Energy Adjustment			Crude			Energy Adjustment									
		Mean	(SD)	Median	Mean	(SD)	Median	Mean	(SD)	Median	Mean	(SD)	Median			Bias, %(95%CI)		95% LOA, %	β	
***Women* (*n* = *122*)**																				
Energy	(kcal)	1990	(511)	1896	1990	(511)	1896	1847	(316)	1817	1847	(316)	1817	0.58	**	105.8	(101.9, 109.9)	69.7, 160.7	0.50	**
Water content	(g)	1588.0	(530.7)	1539.3	1557.0	(422.0)	1479.0	2171.9	(617.4)	2045.4	2134.8	(448.5)	2039.2	0.44	**	71.9	(68.3, 75.8)	40.7, 127.0	0.25	**
Protein	(g)	77.1	(22.9)	75.8	74.7	(11.2)	73.4	69.8	(14.9)	69.7	68.8	(8.7)	68.9	0.36	**	108.1	(103.1, 113.3)	64.4, 181.3	0.41	**
The sum of amino acid residues	(g)	23.7	(8.0)	22.0	23.2	(5.9)	22.9	24.3	(7.4)	23.4	23.8	(5.7)	22.6	0.20	*	96.6	(90.8, 102.7)	49.0, 190.2	0.16	
Total fat	(g)	67.1	(25.0)	64.7	64.1	(13.0)	63.5	64.1	(13.5)	62.0	63.2	(7.5)	61.8	0.34	**	100.2	(94.5, 106.3)	52.5, 191.3	0.74	**
Saturated fatty acids	(g)	20.50	(8.66)	19.33	19.57	(5.02)	19.12	18.82	(4.03)	18.50	18.63	(2.87)	18.51	0.33	**	102.8	(96.3, 109.7)	50.5, 209.1	0.88	**
Monounsaturated fatty acids	(g)	23.88	(10.30)	22.58	22.61	(5.41)	21.98	22.96	(5.51)	22.22	22.58	(3.32)	22.31	0.37	**	98.6	(92.6, 105.1)	49.3, 197.4	0.70	**
Polyunsaturated fatty acids	(g)	13.25	(4.98)	12.59	12.76	(3.28)	12.64	13.02	(3.37)	12.88	12.79	(2.33)	12.28	0.25	**	98.2	(92.6, 104.2)	51.6, 187.0	0.51	**
n-3 polyunsaturated fatty acids	(g)	2.2	(1.2)	1.9	2.2	(1.0)	2.0	2.2	(0.8)	2.0	2.1	(0.6)	2.0	0.38	**	99.4	(93.9, 105.3)	53.3, 185.4	0.40	**
n-6 polyunsaturated fatty acids	(g)	10.9	(4.1)	10.4	10.5	(2.7)	10.5	10.7	(2.7)	10.8	10.5	(2.0)	10.2	0.25	**	98.5	(92.9, 104.4)	51.8, 187.2	0.53	**
Triacylglycerol equivalents	(g)	59.6	(23.4)	56.0	56.6	(11.7)	56.4	56.1	(12.2)	54.2	55.3	(6.9)	54.2	0.33	**	101.0	(94.9, 107.5)	51.2, 199.3	0.74	**
Cholesterol	(mg)	410	(231)	360	389	(176)	364	323	(100)	311	318	(75)	306	0.38	**	115.2	(105.7, 125.5)	45.1, 293.9	0.73	**
Carbohydrate	(g)	254.3	(66.1)	245.7	248.7	(36.0)	246.7	235.0	(44.5)	233.6	232.1	(23.3)	234.4	0.51	**	106.5	(102.4, 110.7)	69.6, 163.0	0.42	**
Total dietary fiber	(g)	16.4	(5.9)	15.2	16.1	(4.8)	15.0	15.3	(5.8)	14.5	15.0	(4.5)	14.0	0.55	**	106.8	(101.3, 112.6)	59.8, 190.5	0.01	
Soluble dietary fiber	(g)	4.0	(1.4)	3.8	4.0	(1.2)	3.9	3.6	(1.3)	3.4	3.6	(1.1)	3.4	0.49	**	107.3	(102.5, 112.4)	65.1, 176.9	0.09	
Insoluble dietary fiber	(g)	11.8	(4.2)	11.0	11.6	(3.4)	10.7	11.1	(4.5)	10.1	10.8	(3.5)	10.1	0.53	**	106.6	(101.0, 112.4)	59.5, 191.1	−0.04	
Ash content	(g)	17.8	(4.6)	17.7	17.6	(3.5)	17.5	17.7	(4.4)	17.6	17.4	(2.8)	17.0	0.45	**	100.4	(96.5, 104.4)	65.4, 154.0	0.09	
Salt equivalent	(g)	9.4	(2.7)	9.1	9.3	(2.4)	9.0	9.6	(2.6)	9.2	9.4	(1.9)	9.2	0.22	*	97.7	(93.0, 102.5)	57.3, 166.6	0.12	
Sodium	(mg)	3716	(1061)	3576	3687	(941)	3565	3789	(1020)	3646	3729	(754)	3623	0.22	*	97.5	(92.4, 102.9)	54.0, 176.1	0.11	
Potassium	(mg)	2783	(954)	2664	2720	(739)	2575	2713	(874)	2545	2653	(637)	2541	0.71	**	101.7	(97.1, 106.5)	61.4, 168.3	0.12	
Calcium	(mg)	601	(253)	560	584	(203)	564	562	(184)	536	553	(161)	523	0.66	**	103.9	(98.1, 110.0)	55.7, 193.9	0.25	**
Magnesium	(mg)	286	(92)	277	280	(67)	266	275	(79)	267	270	(59)	255	0.67	**	103.1	(98.6, 107.8)	63.3, 167.9	0.13	
Phosphorus	(mg)	1141	(344)	1100	1107	(193)	1095	1069	(245)	1056	1053	(164)	1037	0.57	**	104.8	(100.2, 109.7)	64.2, 171.3	0.32	**
Iron	(mg)	9.1	(3.2)	8.7	8.8	(2.2)	8.5	8.5	(2.7)	8.2	8.3	(2.0)	7.8	0.51	**	104.7	(99.9, 109.9)	62.2, 176.5	0.17	
Zinc	(mg)	9.1	(3.4)	8.8	8.8	(1.8)	8.5	8.0	(1.7)	7.8	7.9	(1.0)	7.9	0.26	**	109.2	(104.0, 114.6)	64.3, 185.2	0.61	**
Copper	(mg)	1.27	(0.36)	1.27	1.25	(0.22)	1.20	1.18	(0.31)	1.16	1.17	(0.23)	1.13	0.46	**	103.9	(101.1, 106.7)	77.3, 139.6	0.17	
Manganese	(mg)	3.45	(1.30)	3.39	3.39	(1.06)	3.17	3.83	(3.15)	3.01	3.75	(2.80)	3.02	0.54	**	98.5	(92.8, 104.5)	51.3, 189.2	−0.46	**
Iodine	(μg)	550	(1786)	104	521	(1639)	106	949	(1507)	217	938	(1459)	203	0.15		42.0	(29.5, 59.8)	0.9, 1992.4	−0.13	
Selenium	(μg)	47	(22)	43	46	(19)	43	48	(17)	44	47	(14)	45	0.29	**	94.0	(86.9, 101.6)	40.0, 221.0	0.38	**
Chromium	(μg)	7	(4)	6	7	(3)	6	7	(2)	6	7	(2)	6	0.26	**	101.0	(93.0, 109.7)	41.1, 248.3	0.82	**
Molybdenum	(μg)	168	(61)	164	165	(55)	158	157	(52)	155	155	(48)	146	0.52	**	105.3	(99.1, 111.8)	54.2, 204.4	0.18	
Retinol	(μg)	274	(370)	190	258	(338)	174	233	(224)	179	231	(229)	172	0.14		102.6	(88.4, 119.1)	20.1, 523.4	0.43	**
Retinol equivalents	(μg)	626	(433)	527	607	(394)	507	543	(283)	462	529	(255)	457	0.12		109.1	(98.0, 121.4)	33.7, 353.2	0.33	*
α-Carotene	(μg)	506	(355)	462	504	(347)	462	415	(242)	391	409	(223)	375	0.22	*	101.5	(83.9, 122.7)	12.7, 810.3	0.84	**
β-Carotene	(μg)	3724	(2608)	3036	3666	(2463)	3226	2950	(1762)	2558	2820	(1368)	2586	0.42	**	111.6	(98.3, 126.8)	27.7, 450.7	0.53	**
β-Cryptoxanthin	(μg)	270	(426)	68	274	(470)	66	228	(258)	146	218	(244)	129	0.34	**	76.6	(59.7, 98.3)	5.0, 1172.6	0.38	**
β-Carotene equivalents	(μg)	4176	(2728)	3515	4116	(2580)	3650	3551	(2032)	3114	3405	(1537)	3110	0.35	**	106.4	(94.0, 120.5)	27.3, 414.1	0.51	**
Vitamin D	(μg)	7.7	(6.2)	5.9	7.5	(5.7)	5.7	6.7	(4.6)	5.6	6.5	(4.1)	5.7	0.42	**	104.8	(92.7, 118.6)	27.3, 403.3	0.26	*
α-Tocopherol	(mg)	8.3	(3.4)	7.8	8.0	(2.5)	7.9	8.0	(3.9)	7.3	7.8	(2.8)	7.3	0.34	**	102.1	(96.2, 108.3)	53.4, 195.2	0.13	
β-Tocopherol	(mg)	0.4	(0.2)	0.4	0.4	(0.1)	0.4	0.4	(0.3)	0.4	0.4	(0.3)	0.4	0.09		99.8	(97.0, 102.7)	73.3, 136.0	−0.34	*
γ-Tocopherol	(mg)	11.7	(5.1)	10.7	11.3	(3.9)	11.0	11.1	(3.5)	10.6	10.9	(2.8)	10.3	0.22	*	100.5	(93.7, 107.7)	47.1, 214.4	0.44	**
δ-Tocopherol	(mg)	2.9	(1.4)	2.6	2.9	(1.1)	2.7	2.8	(1.0)	2.7	2.8	(0.9)	2.6	0.28	**	101.0	(95.2, 107.1)	53.2, 191.8	0.38	**
Vitamin K	(μg)	287	(171)	264	281	(157)	252	271	(141)	242	261	(116)	240	0.43	**	100.2	(90.7, 110.7)	33.7, 298.0	0.29	**
Vitamin B1	(mg)	1.02	(0.35)	0.96	1.01	(0.26)	0.99	0.99	(0.23)	0.96	0.98	(0.17)	0.97	0.35	**	101.0	(98.4, 103.7)	76.0, 134.2	0.49	**
Vitamin B2	(mg)	1.44	(0.48)	1.36	1.42	(0.33)	1.39	1.36	(0.36)	1.30	1.35	(0.26)	1.30	0.46	**	102.7	(99.7, 105.7)	74.7, 141.1	0.31	**
Niacin	(mg)	17.9	(6.6)	17.1	17.4	(4.5)	16.6	18.0	(5.5)	17.4	17.7	(3.9)	17.6	0.49	**	97.4	(92.3, 102.8)	54.1, 175.5	0.22	*
Vitamin B6	(mg)	1.34	(0.47)	1.31	1.32	(0.34)	1.26	1.27	(0.41)	1.20	1.26	(0.29)	1.22	0.56	**	102.4	(99.4, 105.5)	74.3, 141.2	0.13	
Vitamin B12	(μg)	7.5	(6.2)	5.4	7.2	(5.1)	5.6	6.4	(3.8)	5.4	6.3	(3.3)	5.7	0.32	**	103.3	(91.7, 116.3)	28.2, 378.9	0.45	**
Folate	(μg)	351	(137)	337	342	(109)	332	371	(151)	340	360	(116)	328	0.56	**	94.7	(89.2, 100.5)	49.4, 181.2	0.02	
Pantothenic acid	(mg)	6.68	(1.96)	6.51	6.52	(1.16)	6.48	6.15	(1.50)	6.07	6.05	(0.94)	5.96	0.46	**	106.3	(102.5, 110.3)	71.3, 158.5	0.23	**
Biotin	(μg)	28.0	(11.2)	25.3	27.4	(9.1)	26.2	28.7	(10.0)	27.1	28.1	(8.3)	26.6	0.53	**	96.2	(90.7, 102.1)	50.6, 183.2	0.15	
Vitamin C	(mg)	102	(59)	88	101	(53)	94	112	(58)	102	107	(43)	96	0.67	**	88.2	(81.9, 94.9)	39.5, 196.7	0.22	**
Ethanol	(g)	6.5	(14.7)	0.5	5.7	(11.8)	0.5	5.3	(11.3)	0.4	4.6	(8.9)	0.7	0.72	**	101.9	(87.5, 118.8)	19.1, 542.8	0.08	
**Median (minimum to maximum)**														**0.38**	**(0.09~0.72)**					

SD, standard deviation; Pearson’s CC, Pearson’s correlation coefficient; bias, the mean difference between methods; 95% CI, 95% confidence interval; LOA, 95% limit of agreement [mean difference ±1.96 *(standard deviation of difference)], expressed as *p* values *: *p* < 0.05 and **: *p* < 0.01. ^a^ 3 d Web24HR: uses the AWARDJP with the web-based 24-h dietary recall survey method. In the entire study period the Web24HR data were collected two or three times. ^b^ 12 d WFR: 12 days of weighted dietary records collected during the entire study period of a year. ^c^ Exponential transform [mean(Web24HR − WFR)] as a ratio of the WFR (all dietary intake data were log-transformed). e.g., 110% indicates overestimation by 10%, and 90% indicates underestimation by 10%. ^d^ The regression slopes of the means of both methods show the differences between the two methods. *p*-values for the the regression slope are shown. When the response and explanatory variables are natural logarithms, a 1% increase in the explanatory variable increases the response variable by β%.

## Data Availability

In adherence to ethical guidelines in Japan, we cannot publicly disclose individual data owing to participant privacy. Furthermore, the obtained informed consent does not include provisions for data sharing. The datasets used in this study can be obtained from the corresponding author upon reasonable request.

## Supplementary Materials

The following supporting information can be downloaded at https://www.mdpi.com/article/10.3390/nu16234140/s1: Table S1-1. Pearson’s correlation coefficients and comparison using the Bland–Altman method between the energy and nutrient intakes of each dietary exposure assessment method and 2 d Web24HR using the 3 d WFR during the same season as a comparison standard (men); Table S1-2. Pearson’s correlation coefficients and comparison using the Bland–Altman method between the energy and nutrient intakes of each dietary exposure assessment method and 2 d Web24HR using the 3 d WFR during the same season as a comparison standard (women); Table S2-1. Pearson’s correlation coefficients and comparison using the Bland-Altman method between the food group intakes of each dietary exposure assessment method and 2 d Web24HR using the 3 d WFR during the same season as a comparison standard (men); Table S2-2. Pearson’s correlation coefficients and comparison using the Bland–Altman method between the food group intakes of each dietary exposure assessment method and 2 d Web24HR using the 3 d WFR during the same season as a comparison standard (women); Table S3. Pearson’s correlation coefficients and comparison using the Bland–Altman method between the energy and nutrient intakes of each dietary exposure assessment method and 2 d Web24HR using the 3 d WFR during the same season as a comparison standard (overall); Table S4. Pearson’s correlation coefficients and comparison using the Bland–Altman method between the food group intakes of each dietary exposure assessment method and 2 d Web24HR using the 3 d WFR during the same season as a comparison standard (overall); Table S5-1. Pearson’s correlation coefficients and comparison using the Bland-Altman method between the food group intakes of each dietary exposure assessment method and 3 d Web24HR using the 12 d WFR as a comparison standard (men); Table S5-2. Pearson’s correlation coefficients and comparison using the Bland–Altman method between the food group intakes of each dietary exposure assessment method and 3 d Web24HR using the 12 d WFR as a comparison standard (women); Table S6. Pearson’s correlation coefficients and comparison using the Bland–Altman method between the energy and nutrient intakes of each dietary exposure assessment method and 3 d Web24HR using the 12 d WFR as a comparison standard (overall); Table S7. Pearson’s correlation coefficients and comparison using the Bland–Altman method between the food group intakes of each dietary exposure assessment method and 3 d Web24HR using the 12 d WFR as a comparison standard (overall); Table S8. Comparison of energy and nutrient intake validity between the 3-day Web24HR and 12-day WFR methods; Table S9. Examination of within- and between-individual variations by energy and nutrients; Table S10. Examination of within- and between-individual variations by food groups; Table S11-1. Pearson’s correlation coefficients and comparison using the Bland–Altman method between the energy and nutrient intakes of each dietary exposure assessment method and 3 d Web24HR using the 12 d WFR as a comparison standard (men/self-administered); Table S11-2. Pearson’s correlation coefficients and comparison using the Bland–Altman method between the energy and nutrient intakes of each dietary exposure assessment method and 3 d Web24HR using the 12 d WFR as a comparison standard (women/self-administered); Table S12-1. Pearson’s correlation coefficients and comparison using the Bland–Altman method between the energy and nutrient intakes of each dietary exposure assessment method and 3 d Web24HR using 12 d WFR as a comparison standard (men/interviewer-administered); Table S12-2. Pearson’s correlation coefficients and comparison using the Bland–Altman method between the energy and nutrient intakes of each dietary exposure assessment method and 3 d Web24HR using the 12 d WFR as a comparison standard (women/interviewer-administered); Table S13. Pearson’s correlation coefficients and comparison using the Bland–Altman method between the energy and nutrient intakes of each dietary exposure assessment method and 3 d Web24HR using the 12 d WFR as a comparison standard (overall/self-administered); Table S14. Pearson’s correlation coefficients and comparison using the Bland–Altman method between the energy and nutrient intakes of each dietary exposure assessment method and 3 d Web24HR using the 12 d WFR as a comparison standard (overall/interviewer-administered); Table S15-1. Pearson’s correlation coefficients and comparison using the Bland-Altman method between the food group intakes of each dietary exposure assessment method and 3 d Web24HR using the 12 d WFR as a comparison standard (men/self-administered); Table S15-2. Pearson’s correlation coefficients and comparison using the Bland–Altman method between the food group intakes of each dietary exposure assessment method and 3 d Web24HR using the 12 d WFR as a comparison standard (women/self-administered); Table S16-1. Pearson’s correlation coefficients and comparison using the Bland–Altman method between the food group intakes of each dietary exposure assessment method and 3 d Web24HR using the 12 d WFR as a comparison standard (men/interviewer-administered); Table S16-2. Pearson’s correlation coefficients and comparison using the Bland–Altman method between the food group intakes of each dietary exposure assessment method and 3 d Web24HR using the 12 d WFR as a comparison standard (women/interviewer-administered); Table S17. Pearson’s correlation coefficients and comparison using the Bland–Altman method between the food group intakes of each dietary exposure assessment method and 3 d Web24HR using the 12 d WFR as a comparison standard (overall/self-administered); Table S18. Pearson’s correlation coefficients and comparison using the Bland–Altman method between the food group intakes of each dietary exposure assessment method and 3 d Web24HR using the 12 d WFR as a comparison standard (overall/interviewer-administered).

## References

[1] Schatzkin A., Subar A.F., Moore S., Park Y., Potischman N., Thompson F.E., Leitzmann M., Hollenbeck A., Morrissey K.G., Kipnis V. (2009). Observational epidemiologic studies of nutrition and cancer: The next generation (with better observation). Cancer Epidemiol. Biomark. Prev..

[2] Willett W. (2013). Nutritional Epidemiology.

[3] Gazan R., Vieux F., Mora S., Havard S., Dubuisson C. (2021). Potential of existing online 24-h dietary recall tools for national dietary surveys. Public Health Nutr..

[4] Subar A.F., Kirkpatrick S.I., Mittl B., Zimmerman T.P., Thompson F.E., Bingley C., Willis G., Islam N.G., Baranowski T., McNutt S. (2012). The Automated Self-Administered 24-hour dietary recall (ASA24): A resource for researchers, clinicians, and educators from the National Cancer Institute. J. Acad. Nutr. Diet..

[5] Simpson E., Bradley J., Poliakov I., Jackson D., Olivier P., Adamson A.J., Foster E. (2017). Iterative development of an online dietary recall tool: INTAKE24. Nutrients.

[6] Meijboom S., van Houts-Streppel M.T., Perenboom C., Siebelink E., van de Wiel A.M., Geelen A., Feskens E.J.M., de Vries J.H.M. (2017). Evaluation of dietary intake assessed by the Dutch self-administered web-based dietary 24-h recall tool (Compl-eat ™) against interviewer-administered telephone-based 24-h recalls. J. Nutr. Sci..

[7] Pendergast F.J., Leech R.M., McNaughton S.A. (2017). Novel Online Or Mobile Methods to Assess Eating Patterns. Curr. Nutr. Rep..

[8] Illner A., Freisling H., Boeing H., Huybrechts I., Crispim S.P., Slimani N. (2012). Review and Evaluation of Innovative Technologies for Measuring Diet in Nutritional Epidemiology. Int. J. Epidemiol..

[9] Timon C.M., van den Barg R., Blain R.J., Kehoe L., Evans K., Walton J., Flynn A., Gibney E.R. (2016). A Review of the Design and Validation of Web- and Computer-Based 24-H Dietary Recall Tools. Nutr. Res. Rev..

[10] Matsumoto M., Murakami K., Yuan X., Oono F., Adachi R., Tajima R., Okada E., Nakade M., Sasaki S., Takimoto H. (2024). A Scoping Review of Dietary Assessment Questionnaires Potentially Suitable for Assessing Habitual Dietary Intake in the National Health and Nutrition Survey, Japan. J. Nutr. Sci..

[11] Nanri A., Shimazu T., Ishihara J., Takachi R., Mizoue T., Inoue M., Tsugane S., JPHC FFQ Validation Study Group (2012). Reproducibility and Validity of Dietary Patterns Assessed by a Food Frequency Questionnaire used in the 5-Year Follow-Up Survey of the Japan Public Health Center-Based Prospective Study. J. Epidemiol..

[12] Tay W., Kaur B., Quek R., Lim J., Henry C.J. (2020). Current Developments in Digital Quantitative Volume Estimation for the Optimisation of Dietary Assessment. Nutrients.

[13] Smith L.P., Ng S.W., Popkin B.M. (2013). Trends in US Home Food Preparation and Consumption: Analysis of National Nutrition Surveys and Time use Studies from 1965–1966 to 2007–2008. Nutr. J..

[14] Orfanos P., Naska A., Trichopoulos D., Slimani N., Ferrari P., van Bakel M., Deharveng G., Overvad K., Tjønneland A., Halkjaer J. (2007). Eating Out of Home and its Correlates in 10 European Countries. the European Prospective Investigation into Cancer and Nutrition (EPIC) Study. Public Health Nutr..

[15] Ministry of Agriculture (2021). F.a.F. FY2020 Summary of the Annual Report on Food, Agriculture and Rural Areas in Japan. https://www.maff.go.jp/j/wpaper/w_maff/r2/pdf/index-1.pdf.

[16] Shinozaki N., Yuan X., Murakami K., Sasaki S. (2021). Development, Validation and Utilisation of Dish-Based Dietary Assessment Tools: A Scoping Review. Public Health Nutr..

[17] Ouchi S., Takachi R., Oda M., Hayashi E., Yamagishi M., Saito Y. (2017). Development and pilot study of a web-based computerized 24-hour dietary recall system-automated web-based assessment system using recipe-data for Japanese: AWARDJP. Res. J. Living Sci..

[18] Hose Y., Ishihara J., Kotemori A., Nakadate M., Maruya S., Tanaka J., Yatsuya H., Aoyama A., Chiang C., Konta T. (2023). Applicability of a Web-based 24-hour Dietary Recall Tool for Japanese Populations in Large-scale Epidemiological Studies. J. Epidemiol..

[19] Kuriyama S., Yaegashi N., Nagami F., Arai T., Kawaguchi Y., Osumi N., Sakaida M., Suzuki Y., Nakayama K., Hashizume H. (2016). The Tohoku Medical Megabank project: Design and mission. J. Epidemiol..

[20] Murakami K., Ishihara J., Takachi R., Sugawara S., Aizawa M., Takahashi I., Obara T., Ishikuro M., Noda A., Ogino M. (2024). Validity and Reproducibility of Food Group Intakes in a Self-Administered Food Frequency Questionnaire for Genomic and Omics Research: The Tohoku Medical Mega-bank Project. J. Epidemiol..

[21] Conway J.M., Ingwersen L.A., Moshfegh A.J. (2004). Accuracy of dietary recall using the USDA five-step multiple-pass method in men: An observational validation study. J. Am. Diet. Assoc..

[22] Conway J.M., Ingwersen L.A., Vinyard B.T., Moshfegh A.J. (2003). Effectiveness of the US Department of Agriculture 5-step multiple-pass method in assessing food intake in obese and nonobese women. Am. J. Clin. Nutr..

[23] Iwaoka F., Yoshiike N., Date C., Shimada T., Tanaka H. (2001). A Validation Study on a Method to Estimate Nutrient Intake by Family Members through a Household-Based Food-Weighing Survey. J. Nutr. Sci. Vitaminol..

[24] (2010). Report of the Subdivision on Resources the Council for Science and Technology Ministry of Education, Culture, Sports, Science, and Technology. Standard Tables of Food Composition in Japan 2010.

[25] (2005). Report of the Subdivision on Resources the Council for Science and Technology Ministry of Education, Culture, Sports, Science, and Technology, Japan. Standard Tables of Food Composition in Japan.

[26] Bland J.M., Altman D.G. (1986). Statistical methods for assessing agreement between two methods of clinical measurement. Lancet.

[27] Bland J.M., Altman D.G. (1999). Measuring agreement in method comparison studies. Stat. Methods Med. Res..

[28] Lombard M.J., Steyn N.P., Charlton K.E., Senekal M. (2015). Application and interpretation of multiple statistical tests to evaluate validity of dietary intake assessment methods. Nutr. J..

[29] Ambrosini G.L., van Roosbroeck S.A.H., Mackerras D., Fritschi L., de Klerk N.H., Musk A.W. (2003). The reliability of ten-year dietary recall: Implications for cancer research. J. Nutr..

[30] Frankenfeld C.L., Poudrier J.K., Waters N.M., Gillevet P.M., Xu Y. (2012). Dietary intake measured from a self-administered, online 24-hour recall system compared with 4-day diet records in an adult US population. J. Acad. Nutr. Diet..

[31] Gregorič M., Zdešar Kotnik K., Pigac I., Gabrijelčič Blenkuš M. (2019). A web-based 24-H dietary recall could be a valid tool for the indicative assessment of dietary intake in older adults living in Slovenia. Nutrients.

[32] Lafrenière J., Laramée C., Robitaille J., Lamarche B., Lemieux S. (2018). Assessing the relative validity of a new, web-based, self-administered 24 h dietary recall in a French-Canadian population. Public Health Nutr..

[33] Wark P.A., Hardie L.J., Frost G.S., Alwan N.A., Carter M., Elliott P., Ford H.E., Hancock N., Morris M.A., Mulla U.Z. (2018). Validity of an online 24-h recall tool (myfood24) for dietary assessment in population studies: Comparison with biomarkers and standard interviews. BMC Med..

[34] Koch S.A.J., Conrad J., Cade J.E., Weinhold L., Alexy U., Nöthlings U. (2021). Validation of the web-based self-administered 24-h dietary recall myfood24-Germany: Comparison with a weighed dietary record and biomarkers. Eur. J. Nutr..

[35] Timon C.M., Blain R.J., McNulty B., Kehoe L., Evans K., Walton J., Flynn A., Gibney E.R. (2017). The development, validation, and user evaluation of Foodbook24: A web-based dietary assessment tool developed for the Irish adult population. J. Med. Internet Res..

[36] Foster E., Lee C., Imamura F., Hollidge S.E., Westgate K.L., Venables M.C., Poliakov I., Rowland M.K., Osadchiy T., Bradley J.C. (2019). Validity and reliability of an online self-report 24-h dietary recall method (Intake24): A doubly labelled water study and repeated-measures analysis. J. Nutr. Sci..

[37] Yuan C., Spiegelman D., Rimm E.B., Rosner B.A., Stampfer M.J., Barnett J.B., Chavarro J.E., Rood J.C., Harnack L.J., Sampson L.K. (2018). Relative Validity of Nutrient Intakes Assessed by Questionnaire, 24-Hour Recalls, and Diet Records as Compared With Urinary Recovery and Plasma Concentration Biomarkers: Findings for Women. Am. J. Epidemiol..

[38] Kato E., Takachi R., Ishihara J., Ishii Y., Sasazuki S., Sawada N., Iwasaki M., Shinozawa Y., Umezawa J., Tanaka J. (2017). Online version of the self-administered food frequency questionnaire for the Japan Public Health Center-based Prospective Study for the Next Generation (JPHC-NEXT) protocol: Relative validity, usability, and comparison with a printed questionnaire. J. Epidemiol..

[39] Fukumoto A., Asakura K., Murakami K., Sasaki S., Okubo H., Hirota N., Notsu A., Todoriki H., Miura A., Fukui M. (2013). Within- and between-individual variation in energy and nutrient intake in Japanese adults: Effect of age and sex differences on group size and number of records required for adequate dietary assessment. J. Epidemiol..

[40] Stote K.S., Radecki S.V., Moshfegh A.J., Ingwersen L.A., Baer D.J. (2011). The number of 24 h dietary recalls using the US Department of Agriculture’s automated multiple-pass method required to estimate nutrient intake in overweight and obese adults. Public Health Nutr..

[41] Zhou H.L., Wei M.H., Cui Y., Di D.S., Song W.J., Zhang R.Y., Liu J.A., Wang Q. (2022). Association between water intake and mortality risk-evidence from a national prospective study. Front. Nutr..

[42] Ma L., Hu Y., Alperet D.J., Liu G., Malik V., Manson J.E., Rimm E.B., Hu F.B., Sun Q. (2023). Beverage consumption and mortality among adults with type 2 dia-betes: Prospective cohort study. BMJ.

[43] Katagiri R., Asakura K., Sasaki S., Hirota N., Notsu A., Miura A., Todoriki H., Fukui M., Date C. (2015). Estimation of habitual iodine intake in Japanese adults using 16 d diet records over four seasons with a newly developed food composition database for iodine. Br. J. Nutr..

[44] Ogawa K., Tsubono Y., Nishino Y., Watanabe Y., Ohkubo T., Watanabe T., Nakatsuka H., Takahashi N., Kawamura M., Tsuji I. (1999). Inter- and intra-individual variation of food and nutrient consumption in a rural Japanese population. Eur. J. Clin. Nutr..

[45] Tokudome Y., Imaeda N., Nagaya T., Ikeda M., Fujiwara N., Sato J., Kuriki K., Kikuchi S., Maki S., Tokudome S. (2002). Daily, weekly, seasonal, within- and between-individual variation in nutrient intake according to four season consecutive 7 day weighed diet records in Japanese female dietitians. J. Epidemiol..

